# Health Tracts

**Published:** 1860-01

**Authors:** 


### HEALTH TRACTS.

A GENTLEMAN of true benevolence has succeeded in accumulating a large fortune within a half-century's time, beginning at the bottom round of the ladder, by following two things: minding his own business and doing good to others. How many there are who would do well to learn that trade! It is simple, useful, and ought not to be hard to learn. What a grand thing it would be for the whole country, just at this juncture, had it learned that useful art!

One of his ways of doing good is to print a multitude of one-paged health tracts at his own expense, and to distribute them personally broadcast. We gave one of them last month, and now another. Like a sensible man, when he can't make a tract to suit himself, he makes a selection from the writings of those who best please him. It will be seen that at this time HALL'S JOURNAL OF HEALTH is his text-book. We commend the extract to every man who has a mind and a conscience. We are constrained to say, however, it strikes us as rather odd that this gentleman should be so mindful and considerate of other people's wives when he persists in not having one for himself:

